# Creating *Effects* in Your Writing—*Tools* to ‘Use’ (or Not)

**DOI:** 10.5334/pme.1540

**Published:** 2024-10-04

**Authors:** Lara Varpio

**Affiliations:** 1 Children’s Hospital of Philadelphia, US

## Abstract

We all want our writing to have impact but realizing that goal is difficult. Prompting specific responses from readers takes savvy and the know-how to wield grammatical tools. This Writer’s Craft addresses the skill of varying emphasis in academic writing through five grammatical tools: italics, bold, dashes, parentheses, and scare quotes. It describes each of these tools, and illustrates when and how to use them effectively.

In the writer’s craft section we offer simple tips to improve your writing in one of three areas: Energy, Clarity and Persuasiveness. Each entry focuses on a key writing feature or strategy, illustrates how it commonly goes wrong, teaches the grammatical underpinnings necessary to understand it and offers suggestions to wield it effectively. We encourage readers to share comments on or suggestions for this section on Twitter, using the hashtag: #how’syourwriting?

## Introduction

Skilled orators bring scripts to life. Volume changes, physical gestures, and dramatic pauses are just some of the tools they use to elicit specific audience reactions. As academic writers, we also want to prompt particular responses from our readers. Sometimes we want readers to take note of specific words. Sometimes we want to problematize terms, highlighting that they don’t mean what the reader might expect. Sometimes we want to focus readers’ attention on a key point of the argument. And sometimes we want to share an interesting point, but just as a tangential side note. Wielding the grammatical tools to convey these meanings takes savvy, but, when mastered, they are handy resources that can add nuance to your academic writing. This Writer’s Craft reviews five grammatical tools for creating effects in your writing—i.e., italics, bold, dashes, parentheses, and scare quotes—and offers advice about when to use them.

## Italics

Italics are used to emphasize a word or phase in a text. When italicized fonts were created in the 15^th^ century, they were intended to “lift certain words out of the surrounding context and mark them as special” [1]. This remains the conventional purpose of italics—to visually accentuate a word (or a collection of words) indicating that it is being distinguished, emphasized, or identified for specific reasons [2]. In scholarly writing, we often see italics used when authors want to identify a particular term that might be unfamiliar to the readers or that might be readily confused with other concepts. This use of italics was artfully employed by Al-Haddad and Lu when they wrote:

Whereas Smith et al. used the term *cultural competence*, and Fruhstorfer et al. used the term *cultural competency*, we use the term *intercultural competence* [3].

Here, italics visually demarcate three terms: cultural competence, cultural competency, and intercultural competence. They draw the reader’s attention to three different terms that could easily be seen as interchangeable given their linguistic similarities (i.e., the words *cultural* and *competence* are key in each term). Italicized text visually indicates to the reader that they are different terms that represent different concepts, each with unique nuanced meanings.

Once a word or phrase is emphasized via italics, authors need not continue to italicize them across the manuscript since they have already been highlighted, and so the reader is aware that they are of particular importance.

## Bold

If italics visually lift words out of a sentence, then bold is equivalent to throwing the words off the page and shining a spotlight on them. There’s nothing subtle about bold text; it is used for in-your-face emphasis. Given its stand-out prominence, it is not surprising that bold text rarely appears in peer-reviewed journals which have traditionally preferred a tone of dry objectivity. In these publications, bold text is generally reserved for headings. It is a tool for visual emphasis that is more commonly used in academic genres where authors are truly trying to stand out from the crowd—e.g., grant submissions.

While uncommon, bold text can be judiciously used in a journal manuscript to emphatically draw the reader’s attention to particularly important words. For example, in Smith et al.’s review, the authors emphasize two kinds of social relationships by bolding key terms:

Research on other marginalized groups has previously noted the protective effect of social **bonds** against the negative psychological and emotional consequences of discrimination. However, in our review, social **bridges** could also significantly reduce the impact of discrimination on refugee doctors [4].

The use of bold font literally makes **bonds** and **bridges** rise high above the other words in the sentence. Research into font design suggests that italics are a more understated form of emphasis than bold [5]. Had Smith et al. used italics instead of bold, the authors’ point about the different nature of social bridges and social bonds would have been more subtly emphasized. Other research warns that some fonts, when bolded, impede readers’ ability to differentiate between letters [6]; this can create confusion and so is a risk that authors should consider. While the gradient of emphasis created by bold vs italic text is likely a matter of taste, authors are well advised to reserve bold text for situations where they wish to create dramatic emphasis.

As with italics, once terms have been emphasized via bold text, there is no need to continue highlighting them across the body of the manuscript.

## Dashes

Another way to create visual emphasis is the use of dashes. Since they are both visually depicted in a sentence as short lines, it is important to distinguish between a hyphen and a dash. A hyphen (i.e. -) is often used to connect words that function together (e.g., Water-repellent material is essential for raincoats.). A dash is created by using two hyphens together (i.e., --) to set off phrases or other content in a sentence (e.g., Her raincoat--a staple of her Malaysian wardrobe--was rarely worn once she moved to Egypt.). In short, the hyphen connects words, while the dash separates segments of text apart from the rest of the sentence. A small caution: most modern word processing programs (e.g., Word) will autocorrect the use of two hyphens into a dash (i.e., a continuous line that is longer than a single dash). This change can be surprising when it first happens, but there is no need to reverse it.

Dashes separate content from the rest of the sentence to highlight the information presented therein. They quite literally interrupt the reader’s progress, drawing attention to the information separated out from the rest of the sentence. They are used to create emphasis, to offer clarity, to present illustrations, or to mark a striking shift in tone or thought [7]. Bynum et al. [8] provide two examples of these purposes. First, to offer an illustration, these authors write:

The nature and effects of shame itself—including intrusive feelings of self-doubt and inadequacy, diminished self-worth, and social isolation—appear to further weaken students’ negotiating power, demanding significant identity work and rendering them more susceptible to the influence of assimilation forces within their environments.

The authors use dashes to offer illustrations of the nature and effects of shame. By being off-set with dashes, the authors’ illustrations are accentuated, stressing one of their study’s central arguments—i.e., that shame has adverse effects on learners.

Second, the authors use the dash to signal both emphasis and a specific tone in the following sentence:

Our study suggests that premedical students, especially those from underrepresented or marginalized backgrounds, may struggle to negotiate their identities within the context of rigid admissions processes and formal structures that impart—explicitly or implicitly—information about who they should be, how they should act, and what they should value.

Here, by separating “explicitly or implicitly” from the rest of the sentence, the authors disrupt the sentence, requiring the reader to pause and note the content placed between dashes. They also create a shift in the sentence’s tone. The authors are not suggesting that admissions processes and formal structures *might* convey messages to premedical students; the authors emphasize that they *do* convey messages and question only if the messaging happens explicitly or implicitly. By using dashes to divide this content away from the rest of the sentence, the authors draw attention to the shift in tone and the point they make via this shift.

## Parentheses

Where dashes draw attention to the separated content, parentheses signal that the enclosed content is less important. The content bounded within parentheses is supplementary information or a minor digression, often an illustration or peripheral point [9]. Schumacher et al. [10] offer an illustration of how parentheses can offer a timely definition of a term:

Effortless deliberate decisions are made when there are sufficient assessment data, the data are mostly congruent (i.e. tell a similar story about the learner) and the data are credible to E/CC members.

The definition of *congruent data* may not be needed since it is rather intuitive, but by offering the just-in-time definition via parentheses they facilitate readers’ understanding.

Since they can enclose long phrases or even whole sentences, it is important to consider how to use punctuation within parentheses. If the enclosed content is a phrase or a single word, no additional punctuation is needed. However, if the enclosed content is a full sentence, then the ending period for that sentence should be embedded within the parentheses.

## Scare Quotes

To signal that a word or phrase is problematic, authors can use scare quotes. Scare quotes are quotation marks that frame a word or set of words indicating that the enclosed word(s) are inaccurate or misleading, odd or inappropriate, or even simply wrong [11]. They emphasize to the reader that the author is calling into question or distancing themselves from the term(s). Konopasky et al. [12] offer a clear illustration of how scare quotes can destabilize the meaning of a term. In their paper, the authors investigate how racialized experiences are hidden in academic writing:

It is more “acceptable” for those experiences to be portrayed through a third-person lens in alignment with the expectations of structured academic discourse than a first-person voice.

Here, the authors clearly do not feel that this practice is “acceptable.” By placing scare quotes around the word, the authors emphasize that they are problematizing this term. The acceptance of the third-person portrayal of Black physicians’ experience is *not* acceptable to these authors. The scare quotes help to ensure that the reader sees that the authors are using the term ironically.

Scare quotes are often misused. Frequently, authors use scare quotes believing that they are emphasizing the enclosed words, not challenging them. This practice should end. As illustrated in this Writer’s Craft, there are several ways for conveying emphasis. In contrast, we do need punctuation that uniquely signals irony and problematic terms.

## When to Use Each Tool

This Writer’s Craft highlights the different purposes of five grammar tools that can create emphasis variation in academic writing. However, four of the five share a purpose of adding emphasis leaving unanswered an important question: How does an author know when to use each tool? Table 1 offers some advice to guide this decision. While this table consolidates suggestions from several grammar handbooks [7,9,13], it summarizes current conventions. There are no definite rules, only style traditions. Like any tradition, it is up to the individual to decide how much they wish to adhere to custom.

**Table 1:** When to Use Different Emphasis Tools.


TOOL	PURPOSES	TYPICAL USAGE	LEVEL OF EMPHASIS

Italics	To distinguish words or short phrases.	With key terms or phrases of various lengths.	Subtle emphasis.

Bold	To dramatically highlight words or phrases.	For individual words or short phrases.	Dramatic emphasis.

Dash	To draw attention to the content between dashes. This content generally offers clarity, presents illustrations, or marks a striking shift in tone or thought.	With long phrases.	Interruptive emphasis.

Parentheses	To present supplementary information or a minor digression.	Often signals a tangential element or an illustration.	De-emphasis.

Scare Quotes	To signal that a term is inaccurate, misleading, odd, or inappropriate.	Often used to indicate that a term is problematic.	Cautionary emphasis.


## Warning: Less is More

When using these tools, remember a cardinal rule: less is more. If an orator recites every line of a script with bold volume changes, wild arm waving, and extended dramatic pauses, we quickly dismiss them as being overly dramatic. Similarly, what separates skilled writers from novices is often the savoir-faire of knowing just how much drama to inject into their texts. Too much, and the writer loses credibility. Just enough, and the writer captures the readers’ attention.

## Conclusion

In summary, italics, bold, dashes, parentheses, and scare quotes are all tools available to elicit effects from readers. As this description illustrates, different tools realize different effects that can bring your writing to life and keep your readers on the edge of their seats. But use these tools sparingly. When overused, everything is emphasized and problematized, and then these tools have defeated their own purposes.
